# DiT-SLAM: Real-Time Dense Visual-Inertial SLAM with Implicit Depth Representation and Tightly-Coupled Graph Optimization

**DOI:** 10.3390/s22093389

**Published:** 2022-04-28

**Authors:** Mingle Zhao, Dingfu Zhou, Xibin Song, Xiuwan Chen, Liangjun Zhang

**Affiliations:** 1 Institute of Remote Sensing and Geographic Information System, Peking University, Beijing 100871, China; zhaomingle@pku.edu.cn (M.Z.); xwchen@pku.edu.cn (X.C.); 2 Robotics and Autonomous Driving Laboratory, Baidu Research, Beijing 100085, China; song.sducg@gmail.com (X.S.); liangjunzhang@baidu.com (L.Z.); 3 National Engineering Laboratory of Deep Learning Technology and Application, Beijing 100085, China

**Keywords:** visual-inertial SLAM, depth estimation, implicit representation, graph optimization, dense mapping

## Abstract

Recently, generating dense maps in real-time has become a hot research topic in the mobile robotics community, since dense maps can provide more informative and continuous features compared with sparse maps. Implicit depth representation (e.g., the depth code) derived from deep neural networks has been employed in the visual-only or visual-inertial simultaneous localization and mapping (SLAM) systems, which achieve promising performances on both camera motion and local dense geometry estimations from monocular images. However, the existing visual-inertial SLAM systems combined with depth codes are either built on a filter-based SLAM framework, which can only update poses and maps in a relatively small local time window, or based on a loosely-coupled framework, while the prior geometric constraints from the depth estimation network have not been employed for boosting the state estimation. To well address these drawbacks, we propose DiT-SLAM, a novel real-time **D**ense visual-inertial SLAM with **i**mplicit depth representation and **T**ightly-coupled graph optimization. Most importantly, the poses, sparse maps, and low-dimensional depth codes are optimized with the tightly-coupled graph by considering the visual, inertial, and depth residuals simultaneously. Meanwhile, we propose a light-weight monocular depth estimation and completion network, which is combined with attention mechanisms and the conditional variational auto-encoder (CVAE) to predict the uncertainty-aware dense depth maps from more low-dimensional codes. Furthermore, a robust point sampling strategy introducing the spatial distribution of 2D feature points is also proposed to provide geometric constraints in the tightly-coupled optimization, especially for textureless or featureless cases in indoor environments. We evaluate our system on open benchmarks. The proposed methods achieve better performances on both the dense depth estimation and the trajectory estimation compared to the baseline and other systems.

## 1. Introduction

Vision-based SLAM systems have been widely explored in the past 20 years and many representative systems have been proposed, which include filter-based approaches (e.g., MonoSLAM [1,2] and the optimization-based approaches (such as PTAM [3], DTAM [4], and ORB-SLAM serials [5,6,7])). Recently, visual-inertial odometry or SLAM methods combined with deep neural networks can achieve more accurate localization results [8,9,10,11], while in the real-time applications, the dominated SLAM approaches are also based on key or corner points extraction and tracking for accurate pose estimation. Furthermore, for building the association between multi-frames in a longtime, a sparse structure map is usually constructed and the bundle adjustment technique is utilized for optimizing the camera pose and the map simultaneously. Although the light-weight sparse map is very suitable for real-time SLAM systems, it also limits its further applications, such as collision-free motion planning, surface-aware AR, or object recognition tasks, due to lack of the dense environment representation. To simultaneously achieve the accurate camera pose estimation and the dense environment reconstructions, many works have been tried, such as LSD-SLAM [12], DynamicFusion [13], and ElasticFusion [14]. However, the former LSD-SLAM system can only give a semi-dense environment reconstruction and the latter two systems are designed specifically for RGB-D cameras in indoor environments.

Direct integration of dense reconstruction (or depth map) into the existing SLAM systems will bring significant computation costs for storing and processing due to its high dimensionality. To address this kind of problem, a compact and optimizable representation has been proposed in reference [15], which compresses the scene depth with a low-dimensional code vector conditioned on the intensity image. Specifically, the dense depth map is transformed into a low-dimensional depth code with the encoder of a CVAE network [16] first. Then, it can be recovered into a dense depth map by a decoder network. Based on this representation, dense reconstruction can be obtained in the SLAM systems. In reference [17], the depth codes are jointly optimized with camera poses and sparse maps by minimizing three objective functions, such as photometric, reprojection, and sparse geometric factors. This strategy can ensure consistency between observations from multiple camera frames. To introduce the absolute scale (or metric) for the monocular SLAM systems, an additional sensor such as the inertial measurement unit (IMU) is often needed. CodeVIO [18] proposes to update depth codes jointly with navigation states in a filter-based sliding window for generating the dense local geometry. However, compared with the filter-based SLAM frameworks, which update the states only in a limited sliding local time window, the graph optimization-based approaches can optimize system states among the selected historical frames and maintain a pose graph or a hierarchical map database [7]. Alternatively, CodeMapping [19] proposes to run the dense mapper thread separately in a loosely-coupled manner to achieve the optimized dense reconstruction. Although the loosely-coupled design makes the system flexible to integrate arbitrary metric information into the system, the loosely-coupled system for local dense mapping can not take advantage of the prior geometric information generated by the depth estimation network, which can provide more geometric constraints for tracking and the graph optimization, especially in textureless or featureless environments.

**Figure 1:**
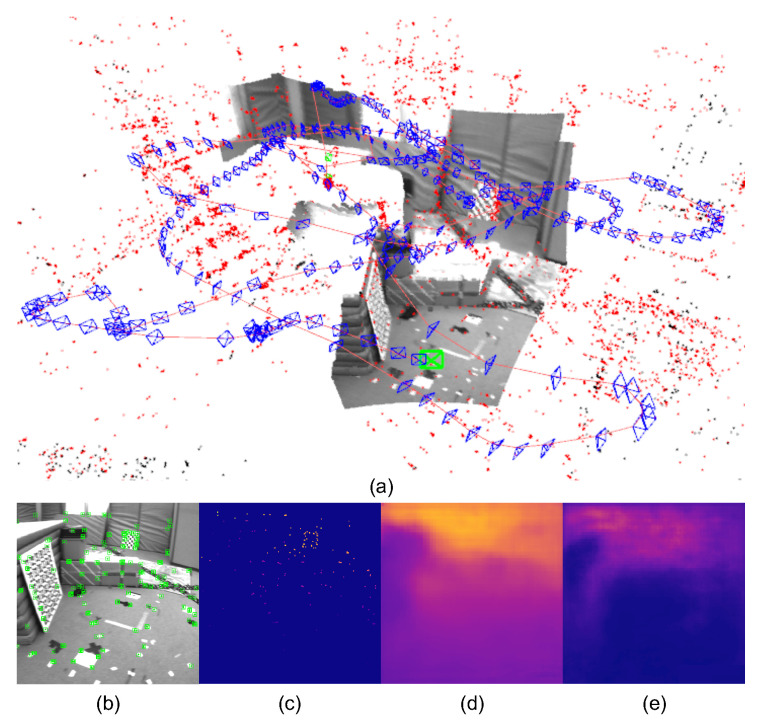
A system demonstration for real-time tracking and local dense mapping on the unseen cross-domain EuRoC dataset of the proposed monocular VI SLAM system. (**a**) Real-time tracking, 3D sparse mapping, and 3D local dense mapping demonstration; (**b**) 2D feature extraction and matching on the gray monocular image; (**c**) the projected sparse depth image; (**d**) the dense depth map predicted by the proposed network only trained on the NYU Depth V2 dataset [20] (warm colors are farther to the camera); (**e**) the depth uncertainty prediction result (warm colors are larger uncertainty).

To address these problems mentioned above, in this paper, we propose DiT-SLAM, a real-time **D**ense visual-inertial (VI) SLAM with **i**mplicit depth representation and **T**ightly-coupled graph optimization. In the proposed system, depth codes can be jointly optimized with camera poses and sparse maps by considering visual, inertial, and depth residuals. In order to achieve real-time performance, we compute the Jacobian of the dense depth map generated from the network with respect to the depth code in a parallel numerical way based on the batch forward on GPUs [18]. In addition, to improve the system’s accuracy and robustness, the spatial and channel attention modules for deep feature refinement and fusion are designed in the depth prediction network. Furthermore, a robust point sampling strategy is also designed to provide geometric constraints to the tightly-coupled optimization for textureless or featureless cases. Using this sampling strategy, depth residuals from featureless regions can supply the prior geometric constraints for both ego-motion tracking and local dense mapping where the feature-based sparse SLAM is prone to be lost or seriously drifted. Figure 1 is a system demonstration for real-time tracking and local dense mapping on the unseen EuRoC dataset of the proposed dense monocular VI SLAM system. In summary, the contributions of our work can be generally summarized as:

1. We develop a real-time dense monocular VI SLAM system based on the tightly-coupled graph optimization by considering visual, inertial, and depth residuals. The proposed VI SLAM system can track the ego-motion of the sensor body and build the local dense map with a metric scale in real-time;
2. A light-weight monocular depth estimation and completion network combined with CVAE and attention mechanisms is proposed. The network can encode and decode a full-resolution dense depth map from a compact representation (i.e., the depth code) of the dense depth image. The network predicts uncertainty-aware dense depth maps in real-time accurately thanks to a light-weight architecture and extremely low-dimensional depth representation (8D depth codes in our system). At the same time, the network shows robustness and generalization capability because of attention modules, leading to improved deep feature refinement and fusion;
3. In order to achieve more consistent local dense mapping and make use of prior geometry information provided by the depth predictions, particularly in featureless environments, we propose a robust point sampling strategy in textureless or featureless cases for depth residuals. This strategy is based on the spatial distribution of feature points. The sampling strategy can provide more constraints from featureless image regions to guide the tightly-coupled optimization for deriving consistent and complete local dense maps, even in the cases that the feature-based sparse SLAM system may lose or drift.

## 2. Related Work

In this section, we discuss the most related monocular visual-inertial SLAM systems, light-weight depth estimation networks, and the depth code-based real-time dense SLAM frameworks.

### 2.1. Monocular Visual-Inertial SLAM

ORB-SLAM2 [6] proposes an effective SLAM system for different types of sensors, such as monocular, stereo, and RGB-D cameras with multi-capabilities, where bundle adjustment and light-weight localization modes are utilized for accurate trajectory estimations. Thus, it can work in real-time on standard CPUs in a wide variety of environments, including indoor sequences of small hand-held or drone devices, cars driving around a city, etc. Meanwhile, ORB-SLAM3 [7] presents a real-time SLAM system that can perform visual, visual-inertial, and multi-map SLAM with monocular, stereo, and RGB-D cameras with maximum a posteriori estimation and place recognition. Besides, VINS-Mono [21] proposes a robust and versatile monocular visual-inertial state estimator, which consists of a camera and a low-cost inertial measurement unit (IMU). A tightly-coupled nonlinear optimization-based method and a loop detection module are employed in VINS-Mono for high-accuracy visual-inertial odometry and better global consistency, thus higher performance can be achieved. Nevertheless, the representative monocular visual-inertial SLAM systems mentioned above, which are based on multi-view geometric methods, can only generate sparse maps with fairly sparse 3D landmarks. These representative monocular visual-inertial SLAM systems are more focused on localization rather than generating a more dense map for other applications, such as obstacle avoidance or indoor 3D reconstruction.

### 2.2. Light-Weight Depth Estimation and Completion Networks

Previous approaches [22,23,24,25] have proven that depth information can be recovered by light-weight deep convolutional neural networks (DCNN)-based frameworks with data-driven strategies. Firstly, the settings that use monocular image and sparse depth as input are also used for dense depth estimation. Refs. [24,25] propose effective frameworks for depth recovery with a monocular image and corresponding sparse depth as input. However, LiDAR or RGB-D sensors are commonly needed, which limit the applications of these methods. Besides, Refs. [22,23] propose efficient and light-weight encoder–decoder architecture for depth prediction with only monocular images as input, which are real-time and applicable to embedded systems. However, the accuracy and reliability of the depth information obtained by current monocular image-based approaches can not be guaranteed, which still restricts the applications of Refs. [22,23]. In fact, the majority of 3D applications are more on visual sequential data instead of separate monocular images and the dense depth is crucial information for 3D geometry inversion from sequential visual data. Therefore, welding depth estimation networks with real-time visual SLAM systems to obtain consistent dense maps has become an interesting research topic recently, and methods on real-time local dense mapping with the compact depth codes present a number of outstanding solutions [15,17,18].

### 2.3. Real-Time Dense SLAM with the Compact Implicit Optimizable Representation

With monocular images as input, compact implicit representation of the dense depth map can be obtained by CodeSLAM [15] with a small number of parameters (the depth code), which is suitable for keyframe-based dense monocular SLAM systems. Global consistency can be achieved by jointly optimizing pose variables and the depth codes of overlapping keyframes. The depth code, in the form of the low-dimensional vector, is encoded from the dense depth image by the encoder of the variational auto-encoder (VAE) network and decoded to recover the dense depth under an auto-encoding scheme in the training phase. Meanwhile, DeepFactors [17], an improved extension from CodeSLAM [15], presents a real-time dense SLAM system with a probabilistic framework that combines a learned compact depth map representation and different types of errors, including photometric, reprojection, and geometric errors. Hence an effective SLAM system with high performance can be achieved. However, DeepFactors [17] only predicts coarse-grained dense depth without a metric scale, since only monocular image data are used without the consideration of the information of sparse points and inertial data. Furthermore, CodeSLAM [15] and DeepFactors [17] are limited by intensive computation due to the quit-heavy-weight network architecture and Jacobian calculation with the auto-gradient method in deep learning libraries. CodeVIO [18] presents an effective real-time SLAM system that consists of a light-weight DCNN-based depth estimation network and a tightly-coupled visual-inertial odometry (VIO) system. At the same time, CodeVIO can provide accurate state estimates and local dense depth maps of the immediate surroundings. Besides, only sparse measurements of depth are updated in the depth code of Ref. [18] with a parallel Jacobian computation method, but CodeVIO [18] is based on the filtering update, which can not estimate a larger number of points and poses in a tightly-coupled graph optimization from the co-visibility graph rather than in a small time window. In addition, it consists of a fairly simple light-weight network leading to a weak robustness and generalization capability of the depth prediction, especially on an unseen cross-domain dataset. Leveraging a compact scene representation, CodeMapping [19] proposes a novel dense mapping framework for sparse visual SLAM systems, where the camera trajectory and locations of landmarks can be accurately and reliably estimated, thus they can be used not only for local mapping but also globally consistent dense 3D reconstruction. As a result of the dense mapping thread in CodeMapping [19] under a loosely-coupled manner, the prior depth estimation generated from the network can not provide more geometric constraints to improve the tacking results simultaneously. Thus, the tightly-coupled optimization is performed in our proposed system, guiding the optimization to improve pose and sparse map estimation, particularly in challenging environments, such as white walls.

## 3. System Overview

**Figure 2:**
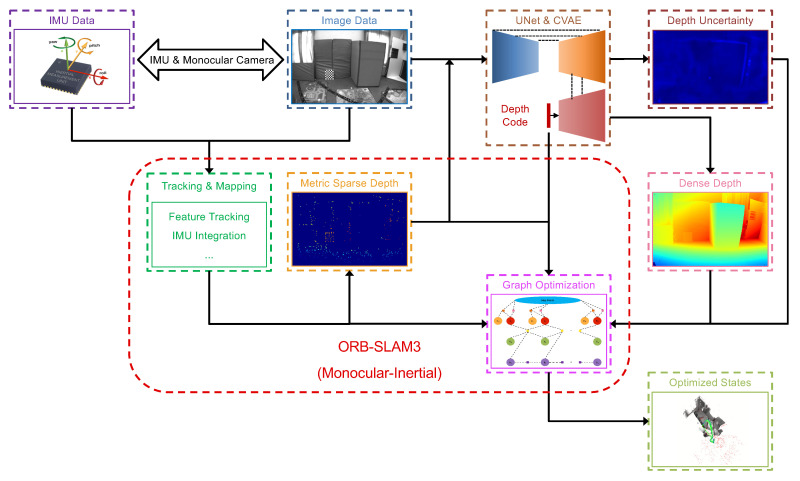
System overview of our proposed framework. The system is based on ORB-SLAM3 in the monocular visual-inertial modality with a tightly-coupled back-end based on graph optimization by considering visual, inertial, and depth residuals. RGB/gray images and projected sparse depth images derived from the geometric SLAM are taken as the inputs for the network encoder.

The proposed system is a tightly-coupled monocular visual-inertial SLAM based on the ORB-SLAM3 [7] by integrating dense depth completion and local dense mapping modules additionally. Compared to the ORB-SLAM3, our system can provide local dense 3D maps in real-time with low-dimensional depth codes. Specifically, the proposed system contains three main components: a sparse features-based monocular visual-inertial SLAM framework, a light-weight depth completion network, and a tightly-coupled graph optimization module for local dense mapping. A general pipeline of our proposed system is illustrated in Figure 2.

### 3.1. Monocular Visual-Inertial SLAM with Sparse Maps

We take the ORB-SLAM3 with the monocular visual-inertial (MVI) mode as the framework by taking a monocular camera and an IMU data sequence as inputs. The MVI modality is an ORB feature-based tightly-integrated SLAM system that is completely derived from the maximum a posteriori (MAP) state estimation. The ORB-SLAM3 shows significantly more accurate and robust results on tracking and sparse mapping, especially in indoor environments [7] with the multi-map data association and robust visual-inertial fusion techniques.

The MVI system takes the image (RGB or gray) and IMU data for ego-motion estimation and a sparse local map reconstruction. For tracking, the system solves a visual-inertial optimization problem for the state variables using the last two frames while keeping the sparse local map unchanged. After obtaining the tracking observations and their corresponding sparse points, the state variables are optimized by the local mapping thread with the keyframes in a sliding window [7]. At the same time, local mapping optimization also includes observations from co-visible keyframes with fixed poses of the sparse map points. Except for the state estimation on body poses and sparse maps, IMU preintegration [26,27] within optimization contributes to derive a projected scale-metric sparse depth image, which is taken as an input channel of the light-weight depth completion network.

### 3.2. Light-Weight Depth Completion Networks with Attention Mechanisms

**Figure 3:**
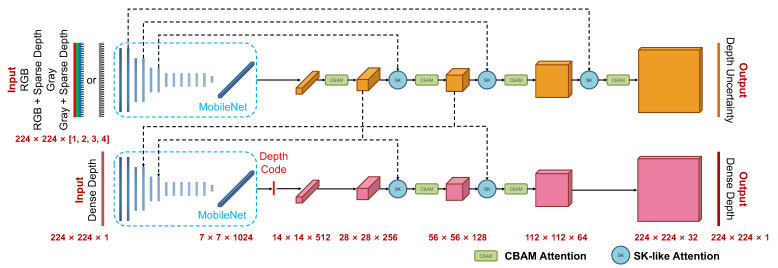
The architecture of our proposed light-weight network with attention mechanisms for uncertainty-aware dense depth estimation and completion. The network supports multiple input modalities: RGB, RGB + Sparse Depth, Gray, and Gray + Sparse Depth. The top stream is a UNet network to extract input features and outputs the dense depth uncertainty map. The bottom stream is the CVAE network conditioned on the features from the above UNet and predicts the dense depth map.

To obtain local dense maps in real-time, we leverage a light-weight depth estimation and completion network based on FastDepth [23] and the conditional CVAE. The depth estimation network can support various input modalities, such as RGB, RGB + sparse depth, gray, and gray + sparse depth. Except for the dense depth estimation, it can also produce the uncertainty of the depth estimation. As shown in Figure 3, we use the light-weight MobileNet [28] as the backbone, which takes advantage of depth-wise separable convolutions with real-time performance. Furthermore, in order to improve the accuracy of depth estimation with the more low-dimensional depth code than in previous works, we introduce two types of light-weight attention modules as convolutional block attention modules (CBAM) [29] and selective kernel (SK) modules [30]. They are employed to refine deep feature extraction and feature fusion, respectively, for improving the accuracy and robustness of depth prediction. More details are described in Section 4.

### 3.3. Tightly-Coupled Graph Optimization with Depth Residuals

For visual-inertial SLAM with sparse maps, the system state vectors to be optimized can be noted as:

(1) $$S_{i}\dot{=}\left\{T_{i},v_{i},b_{i}^{g},b_{i}^{a}\right\}$$

where $T_{i}=\left[R_{i},p_{i}\right]\in \mathrm{SE}\left(3\right)$, $v_{i}$, $b_{i}^{g}$, $b_{i}^{a}$ is the sensor body pose, velocity in the world reference system, gyroscope, and accelerometer biases of the frame in timestamp *i*, respectively. In addition, to attain the dense mapping tightly integrated with the whole system, the proposed system considers taking the latent depth codes of selected keyframes as system states in the back-end optimization. For these selected keyframes with optimizable depth codes, the state vectors in our system can be expressed as:

(2) $$S_{k}\dot{=}\left\{T_{k},v_{k},b_{k}^{g},b_{k}^{a},c_{k}\right\}$$

where $c_{k}\in R^{C\times 1}$ is the *C*-dimensional depth code of the corresponding keyframe and is the input of the decoder of the CVAE stream in the proposed light-weight network to generate a dense depth map of the keyframe. Specifically, the depth code in our system is a vector with the extremely low dimension C=8. In order to achieve local dense mapping in real-time, the depth residuals from fixed sparse map points in tracking and local mapping modules and the parallel finite difference method for the numerical computation of the full Jacobian of the predicted dense depth map with respect to the depth code vector through the CVAE decoder inspired by CodeVIO [18] are applied in the back-end optimization. We describe the module in detail in Section 5.

## 4. Light-Weight Depth Estimation and Completion Network with Attention Mechanisms

Figure 3 shows the architecture of the proposed light-weight network for depth estimation and completion with latent depth codes. In order to achieve real-time performance in depth prediction, all convolution layers of the network are depth-wise separable. An efficient MobileNet with depth-wise decomposition serves as the encoders in both the UNet stream (top stream) for feature extraction and the CVAE stream (bottom stream) for dense depth auto-encoding. The upsampling layers leverage depth-wise separable convolutions with a kernel size of five and nearest-neighbor based interpolation in the decoders. In the CVAE stream, during the training phase, the bottom stream takes dense depth as input for transforming the dense depth to a low-dimensional depth code first and then decodes the depth code to a dense depth conditioned on the image features extracted from the top stream. During the online inference, with the trained model, the network predicts the dense depth map from a depth code sampled from the standard normal distribution [31]. The sampled depth code passes the decoder of CVAE conditioned on the features from the top UNet stream, and the encoder of the bottom CVAE stream is not needed since the dense depth maps of keyframes are unknown in online inference. The architecture of the trained network in the online inference phase is shown in Figure 2, where the VAE encoder is ignored. Light-weight attention modules are used for deep feature refinement and fusion, leading to the more accurate depth prediction from more low-dimensional depth codes. Shorter depth codes can achieve fast inference and graph optimization with lower latency.

The network is designed for multiple input modalities of the RGB image, the RGB image concatenated with a sparse depth map, the gray image, and the gray image concatenated with a sparse depth map. As shown in Figure 3, the input dimensions of UNet stream for different modalities mentioned above are 224×224×3, 224×224×4, 224×224×1, and 224×224×2, respectively. For a trained network model used in the visual-inertial SLAM system, we exploit the RGB or gray image stacked with a sparse depth map modality to make use of the information of sparse map points with a metric scale projected from the SLAM sparse maps. Notably, although attention modules will bring a slightly additional computation burden, the inference time of the proposed network is close to that of the network in CodeVIO because of the low-dimensional depth code.

### 4.1. Deep Feature Extraction with CBAM Attention Modules

To refine the feature extraction in the network, the effective attention modules for feed-forward convolutional neural networks called convolutional block attention modules (CBAM) [29] are utilized for adaptive feature refinement with a negligible increment of computation. A CBAM attention block consists of the channel and spatial attention modules. The channel attention exploits the inter-channel relationship of features and focuses on extracting the meaningful deep features from a given input image. Spatial attention makes use of the inter-spatial relationship of deep features. Different from channel attention, the spatial attention focuses on selecting the informative part of features, which can be complementary to channel attention. Given an intermediate feature map $F\in R^{H\times W\times C}$ as input, CBAM sequentially infers a 1D channel attention map $M_{c}\in R^{1\times 1\times C}$ and a 2D spatial attention map $M_{s}\in R^{H\times W\times 1}$. The overall attention process can be summarized as:

(3) $$\begin{array}{cc} \; & F_{c}=M_{c}\left(F\right)⊗F\\ \; & F_{s}=M_{s}\left(F_{c}\right)⊗F_{c}\\ \; & F_{\mathrm{refined}}=F_{s}⊕F \end{array}$$

where ⊗ and ⊕ denote tensor element-wise multiplication and addition, respectively, and $F_{\mathrm{refined}}$ is the final refined output feature map passed by the CBAM block. We integrate CBAM attention blocks in the decoders of UNet and CVAE streams as a residual pass way fused with input features.

### 4.2. Deep Feature Fusion with SK-like Attention Modules

Inspired by selective kernel networks (SK) [30], SK-like units take the place of the addition operation to fuse feature maps via *Fuse* and *Select* operators from two different branches (previous convolutional layers and skip connections) adaptively using softmax [32] attention, which is guided by the information in these branches. We regard two feature maps from previous convolutional layers and skip connections as features from two branches with different kernel sizes mentioned in the original SK implementation [30]. Given two feature maps from two branches $F_{1},F_{2}\in R^{H\times W\times C}$ with the same shapes, firstly, we *fuse* these feature maps via an element-wise summation, the global average pooling for channel-wise information, and the guidance feature generating:

(4) $$\begin{array}{cc} \hat{F} & =F_{1}⊕F_{2}\\ S_{c}=\mathrm{AVG}\left({\hat{F}}_{c}\right) & =\frac{1}{H\times W}\sum_{i=1}^{H}\sum_{j=1}^{W}{\hat{F}}_{c}\left(i,j\right)\\ Z & =\mathrm{FC}(S) \end{array}$$

where $S_{c}$ and ${\hat{F}}_{c}$ are the channel-wise map in channel *c* of S and $\hat{F}$, respectively, AVG(·) is the average pooling through spatial dimensions H×W, and FC(·) is a simple fully connected (FC) layer to generate a compact feature $Z\in R^{d\times 1}$ to enable the guidance for the precise and adaptive selections next. In *Select*, soft-attention-across channels are used to adaptively select different spatial information, which is guided by the compact feature Z. Specifically, we employ the softmax operator in the channel dimension to generate the soft attention and finally fuse the input feature maps:

(5) $$\begin{array}{cc} {\left[A_{1},A_{2}\right]}^{T} & =Softmax({\left[{\mathrm{FC}}_{1}\left(Z\right),{\mathrm{FC}}_{2}\left(Z\right)\right]}^{T})\\ F_{\mathrm{fused}} & =A_{1}F_{1}⊕A_{2}F_{2} \end{array}$$

where $F_{\mathrm{fused}}$ is the fused feature map, ${\mathrm{FC}}_{1}\left(\cdot \right)$ and ${\mathrm{FC}}_{2}\left(\cdot \right)$ are fully connected layers to map the compact feature Z into the original input channels for $F_{1},F_{2}$. $A_{1}$, $A_{2}$ are the soft attention for input features $F_{1},F_{2}$, respectively. As described in Section 5 and Section 6, SK-like units can achieve more accurate and robust feature selection and fusion with an ignorable increment of computation and latency because of the light-weight network architecture and the parallel numerical Jacobian computation.

### 4.3. Loss Functions

In the training phase, the loss function is the summation of the uncertainty-aware dense depth reconstruction loss and the KL divergence (KLD) loss [31] for the CVAE part. The uncertainty-aware depth reconstruction loss evaluates the negative log-likelihood of the predicted dense depth and forces the network to attenuate the loss of difficult regions and to focus on reconstructing parts that can be well explained [15]. The learned uncertainty can also serve to gauge the reliability of the dense depth reconstruction. The CVAE KL divergence loss tries to enforce the depth code manifold distribution to be close to a standard zero-mean Gaussian distribution. The network outputs a dense depth map and an uncertainty map. The uncertainty-aware dense depth reconstruction loss is:

(6) $$\begin{array}{c} L_{rec}=\frac{1}{\left|\Omega \right|}\sum_{x\in \Omega }\frac{|{\hat{D}}_{rec}\left(x\right)-D_{GT}\left(x\right)|}{\hat{B}\left(x\right)}+log\left(\hat{B}\left(x\right)\right) \end{array}$$

where Ω is the set of all valid pixels in the input dense depth of ground truth $D_{GT}$. ${\hat{D}}_{rec}$ and $\hat{B}$ are the predicted dense depth and uncertainty, respectively. The KL divergence loss is:

(7) $$\begin{array}{c} L_{KLD}=\frac{1}{2}\sum_{i}\left[\mu_{i}^{2}+\sigma_{i}^{2}-log\left(\sigma_{i}^{2}\right)-1\right] \end{array}$$

where $\mu_{i}$ and $\sigma_{i}$ are the encoded mean and standard deviation of the predicted distribution from the output of the CVAEs encoder. Then, the total loss function of the proposed network in training can be noted as:

(8) $$\begin{array}{c} L_{total}=L_{rec}+L_{KLD} \end{array}$$

## 5. Tightly-Coupled Graph Optimization with Depth Residuals

### 5.1. Visual and Inertial Residuals

For the optimization problem in the SLAM system, visual residuals are defined as the reprojection errors $r_{ij}$ related to the image frame *i* and the 3D map point *j* at the position $x_{j}$. Therefore, the visual residual in the form of the reprojection error is [7]:

(9) $$\begin{array}{c} r_{ij}=u_{ij}-\Pi \left(T_{bc}^{-1}T_{i}^{-1}⊞x_{j}\right) \end{array}$$

where Π(·) is the projection function for the corresponding camera model, $u_{ij}$ is the observation of point *j* at the image of frame *i* with an observation covariance matrix $\Sigma_{ij}$, $T_{bc}\in \mathrm{SE}(3)$ represents the rigid reference transformation from the camera frame to the IMU sensor body, and ⊞ is the transformation operation of the SE(3) group over $R^{3}$ elements. In a visual-inertial SLAM system, IMU preintegration measurements [26] can be obtained by the measurement integration between consecutive image frames, *i* and i+1, for rotation, velocity, and position with the formulation on manifolds [27], noted as $\Delta R_{i,i+1}$, $\Delta v_{i,i+1}$, and $\Delta p_{i,i+1}$, respectively:

(10) $$\begin{array}{cc} \Delta R_{i,i+1} & =\prod_{k=i}^{i+1-\delta t}\mathrm{Exp}\left(\left({\tilde{\omega }}_{k}-b_{k}^{g}-\eta_{k}^{gd}\right)\delta t\right)\\ \Delta v_{i,i+1} & =\sum_{k=i}^{i+1-\delta t}\Delta R_{i,k}\left({\tilde{a}}_{k}-b_{k}^{a}-\eta_{k}^{ad}\right)\delta t\\ \Delta p_{i,i+1} & =\sum_{k=i}^{i+1-\delta t}\left(\Delta v_{i,k}\delta t+\frac{1}{2}\Delta R_{i,k}\left({\tilde{a}}_{k}-b_{k}^{a}-\eta_{k}^{ad}\right){\delta t}^{2}\right) \end{array}$$

where ${\tilde{\omega }}_{k}$, $b_{k}^{g}$, and $\eta_{k}^{gd}$ are the sensor measurement, the slowly varying sensor bias, and the additive white noise for the gyroscope in discrete time, and ${\tilde{a}}_{k}$, $b_{k}^{a}$, and $\eta_{k}^{ad}$ are for accelerometer, respectively; δt is the discrete time step for IMU measurements [27]. For the system states $S_{i}$ and $S_{i+1}$ in Equation (1) or Equation (2), the inertial residual between consecutive frames $r_{I_{i,i+1}}$ with a covariance matrix $\Sigma_{I_{i,i+1}}$ for the whole measurement vector can be derived from preintegration as [7]:

(11) $$\begin{array}{cc} r_{I_{i,i+1}} & =[r_{\Delta R_{i,i+1}},r_{\Delta v_{i,i+1}},r_{\Delta p_{i,i+1}}] \end{array}$$

where $r_{\Delta R_{i,i+1}}$, $r_{\Delta v_{i,i+1}}$, and $r_{\Delta p_{i,i+1}}$ are the component of the inertial residual for rotation, velocity, and position, respectively:

(12) $$\begin{array}{cc} r_{\Delta R_{i,i+1}} & =\mathrm{Log}(\Delta R_{i,i+1}^{T}R_{i}^{T}R_{i+1})\\ r_{\Delta v_{i,i+1}} & =R_{i}^{T}\left(v_{i+1}-v_{i}-g\Delta t_{i,i+1}-\Delta v_{i,i+1}\right)\\ r_{\Delta p_{i,i+1}} & =R_{i}^{T}\left(p_{j}-p_{i}-v_{i}\Delta t_{i,i+1}-\frac{1}{2}g\Delta t_{i,i+1}^{2}\right)-\Delta p_{i,i+1} \end{array}$$

where the function Log(·) maps variables from the Lie group to the real 3D vector space.

### 5.2. Depth Residuals: Geometry and Consistency

In order to tightly optimize the results of local dense mapping with visual-inertial states online, the system needs to introduce the residuals related to depth representations of keyframes. Optimization on every pixel of the depth image in a naive way, obviously, is computationally redundant and impractical for a real-time SLAM system. From the light-weight depth completion network described in Section 4, we can obtain a compact and optimizable low-dimensional depth representation of selected keyframes to achieve the tightly-coupled optimization with visual and inertial state variables for dense mapping. The keyframe with a depth code initialized from the zero-mean normal distribution can infer a dense depth image using the light-weight network (Section 4), where the encoder of the bottom CVAE stream is ignored in the online phase. The predicted dense depth image $d_{k}=F\left(I_{k},S_{k},c_{k}\right)$ can be formulated as a function of the RGB/gray image $I_{k}$, projected sparse depth image $S_{k}$ from the valid 3D map points, and the depth code $c_{k}$ of the keyframe *k* through the network. For a keyframe with a corresponding depth code, there are two depth residuals related to the predicted dense depth image that can be formulated on sparse points of the keyframe [18]. The depth $z_{kj}$ derived from projecting a key point in 3D position $x_{j}$ to the keyframe *k* at the pixel coordinate $P^{j}={\left[p,q\right]}^{T}$ and the depth from the predicted dense depth image $d_{k}\left[p,q\right]$ can form the geometric depth residual $r_{kj}$:

(13) $$\begin{array}{cc} r_{kj} & =z_{kj}-d_{k}\left[p,q\right]\\ \; & ={\left[T_{bc}^{-1}T_{k}^{-1}⊞x_{j}\right]}_{z}-F\left(I_{k},S_{k},c_{k}\right)\left[p,q\right] \end{array}$$

where ${\left[\cdot \right]}_{z}$ extracts the *z* component of the transformed vector.

Besides, the depth of the corresponding projected points should be consistent among keyframes from different views. To jointly refine the consistency of the reconstructed geometry, the system formulates the depth consistency residual, which gauges the pixel-wise difference between the dense depth maps of sampled sparse points after warping them to a uniform reference frame. As for two different keyframes $k_{1}$, $k_{2}$ with depth codes $c_{k_{1}}$, $c_{k_{2}}$ and a corresponding point *j* at the 2D image coordinates $P_{1}^{j}={\left[p_{1},q_{1}\right]}^{T}$, $P_{2}^{j}={\left[p_{2},q_{2}\right]}^{T}$ of $k_{1}$, $k_{2}$, the depth consistency residual $r_{k_{1}k_{2}}^{j}$:

(14) $$\begin{array}{cc} r_{k_{1}k_{2}}^{j} & =d_{k_{1}}\left(P_{1}^{j}\right)-{\left[T_{k_{1}k_{2}}⊞\Pi^{-1}\left(P_{2}^{j},d_{k_{2}}\left(P_{2}^{j}\right)\right)\right]}_{z}\\ \; & =F\left(I_{k_{1}},S_{k_{1}},c_{k_{1}}\right)\left[p_{1},q_{1}\right]-\\ \; & \;\;\;\;{\left[T_{k_{1}k_{2}}⊞\Pi^{-1}\left(P_{2}^{j},F\left(I_{k_{2}},S_{k_{2}},c_{k_{2}}\right)\left[p_{2},q_{2}\right]\right)\right]}_{z} \end{array}$$

where $T_{k_{1}k_{2}}$ is the transformation from $k_{2}$ to $k_{1}$, $\Pi^{-1}\left(\cdot \right)$ is the inverse projection function for the camera model.

**Figure 4:**
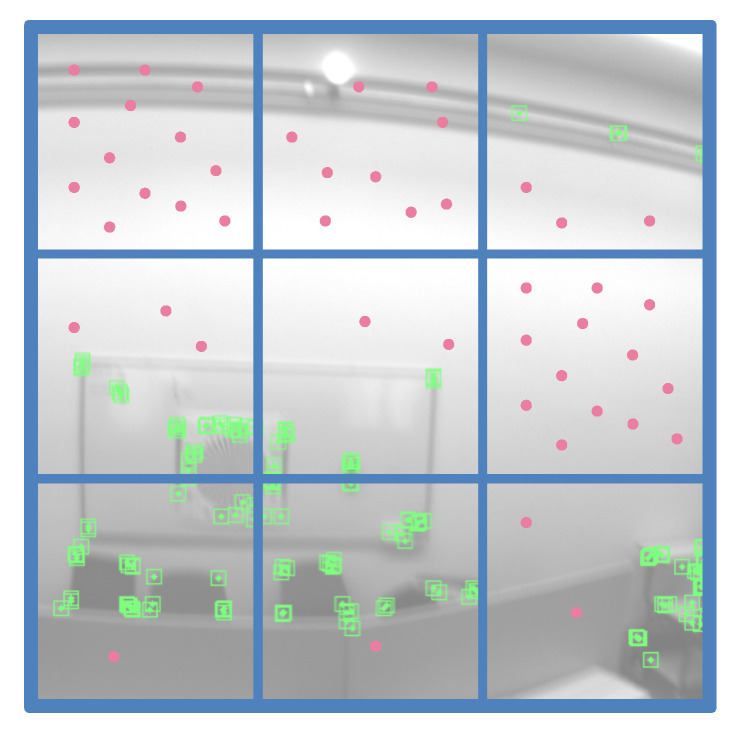
Illustration of the robust point sampling strategy for depth consistency residuals. The sampling method is based on the spatial distribution of 2D feature points in the divided image cells (3×3) and is inclined to sample more points from the featureless regions (green points are 2D feature points extracted by SLAM system for feature matching; red points are sampled points for depth consistency residuals). Notice that the depth consistency residuals are based on the randomly sampled points rather than 2D feature points.

For depth consistency residuals, we propose a sparse point sampling strategy to provide constraints in textureless or featureless regions for local dense mapping (Figure 4). We can divide the keyframe image into nine (3×3) cells and compute the spatial distribution of extracted 2D feature points. According to the number distribution of the feature points among the nine cells, we can maintain a weight queue $Q_{w}=\left\{q_{l}|l=1,\ldots ,9\right\}$ to measure the normalized relative amount of feature points, which every cell contains ($\sum_{l=1}^{9}q_{l}=1$). We can reversely sort the queue $Q_{w}$ and obtain the corresponding reverse queue $Y_{w}=\left\{\gamma_{l}|l=1,\ldots ,9\right\}\;\left(\sum_{l=1}^{9}\gamma_{l}=1\right)$. Given the number of points of a keyframe for the depth consistency residual $N_{consis}$, the uniformly sampled point number $n_{consis}$ in the cell *l* can be derived using $Y_{w}$: $n_{consis}=\gamma_{l}N_{consis}$. For these featureless patches without enough feature points, we uniformly sample more image pixels in that patch for optimization. As is shown in Figure 4, the proposed sampling strategy is based on the spatial cellular distribution of extracted 2D feature points in the divided image cells (3×3) and is inclined to sample points from the featureless regions. Therefore, depth consistency residuals can provide more constraints in textureless or featureless regions for local dense mapping (Section 6). This can improve the robustness of the SLAM system in challenging environments, such as a white wall. Depth consistency residuals, which tend to capture geometric constraints in the featureless regions, can provide more geometry information to improve tracking accuracy and robustness under a tightly-coupled manner, particularly in textureless or featureless environments where sparse feature-based SLAM systems are prone to be lost or seriously drifted.

In general feature-based visual SLAM methods, the correspondence of points is from feature matching. In the depth consistency residuals of the proposed method, the point correspondence is directly derived from the motion transformation to minimize the error of predicted depths. It is similar to the direct method-based SLAM frameworks [12,33], which minimize the photometric error with the correspondence from the direct motion transformation.

### 5.3. Tightly-Coupled Graph Optimization with the Numerical Parallel Jacobian Computation

**Figure 5:**
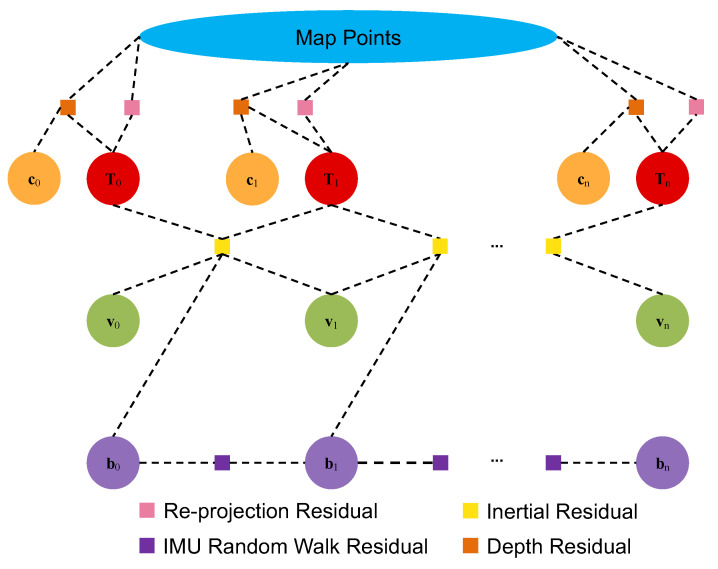
Factor graph of the system considering visual reprojection residuals, inertial residuals, IMU random walk residuals, and depth residuals on sparse points from the output of the depth completion network. $T_{n}$: pose states, $v_{n}$: velocity states, $b_{n}$: IMU bias states, $c_{n}$: depth codes.

Therefore, the optimization problem of the system can be posed as a keyframe-based minimization problem combining visual, inertial, and depth residual terms [34]. For a given set KF of *m* keyframes within the sliding-window with a set of corresponding states $S_{m}\dot{=}\left\{S_{0},\ldots ,S_{m-1}\right\}$, a set of *n* 3D map point states $X_{n}\dot{=}\left\{x_{0},\ldots ,x_{n-1}\right\}$, and a set of the compact depth codes of selected $m^{\prime }$ ($0<m^{\prime }<m$) keyframes $C_{m^{\prime }}\dot{=}\left\{c_{0},\ldots ,c_{m^{\prime }-1}\right\}$, the minimization formula of the optimization problem can be stated as:

(15) $$\begin{array}{cc} \min_{S_{m},X_{n},C_{m^{\prime }}}( & \sum_{i=0}^{m-2}\left|\right|r_{I_{i,i+1}}{\left|\right|}_{\Sigma_{I_{i,i+1}}^{-1}}^{2}+\\ \; & \sum_{j=0}^{n-1}\sum_{i\in {\mathrm{KF}}^{j}}\rho_{\mathrm{Huber}}\left(|\right|r_{ij}{\left|\right|}_{\Sigma_{ij}^{-1}})+\\ \; & \sum_{j=0}^{n^{\prime }-1}\sum_{k\in {\mathrm{KF}}^{j}}\rho_{\mathrm{Huber}}\left(|\right|r_{kj}{\left|\right|}_{\Sigma_{kj}^{-1}})+\\ \; & \sum_{j=0}^{n^{\prime \prime }-1}\sum_{\begin{array}{c} k_{1},k_{2}\in {\mathrm{KF}}^{j}\\ k_{1}\neq k_{2} \end{array}}\left|\right|r_{k_{1}k_{2}}^{j}{\left|\right|}_{\Sigma_{k_{1}k_{2}}^{j^{-1}}}^{2}) \end{array}$$

where $\rho_{\mathrm{Huber}}\left(\cdot \right)$ is the robust kernel function with Huber kernel [35] to reduce the influence of outliers for $r_{ij}$ and $r_{kj}$ owing to the spurious matching on key points, $n^{\prime }$ is the configurable number of selected map points for $r_{kj}$, $n^{\prime \prime }$ is the sample number of image points for $r_{k_{1}k_{2}}^{j}$, ${\mathrm{KF}}^{j}$ is the keyframe subset of KF, which have the observations to the point *j*, $\Sigma_{kj}$ and $\Sigma_{k_{1}k_{2}}^{j}$ are the corresponding covariance matrices fetched and propagated at the coordinates from the predicted depth uncertainty map, respectively. Here, apart from the graph optimization conducted by the basic sparse SLAM framework, a sliding-window of the five most co-visible keyframes selected by the co-visibility graph is employed to the optimization, introducing the depth residuals for local dense mapping. Figure 5 shows that the system assembles a tightly integrated back-end based on the factor graph optimization, including visual, inertial, and depth residuals for real-time tracking and local dense mapping.

The system utilizes the the Levenberg–Marquardt algorithm [36,37] to solve the optimization problem in the back-end. The depth residuals in Equations (13) and (14) involve predicted dense depth maps related to depth codes. To optimize the depth code states, the system requires the Jacobians of depth residuals with respect to depth codes:

(16) $$\begin{array}{cc} J_{c_{k}}^{r_{kj}} & =\frac{\partial r_{kj}}{\partial c^{T}}{|}_{c=c_{k}}=\frac{\partial r_{kj}}{\partial F}\frac{\partial F}{\partial c^{T}}{|}_{c=c_{k}}\\ J_{c_{k}}^{r_{k_{1}k_{2}}^{j}} & =\frac{\partial r_{k_{1}k_{2}}^{j}}{\partial c^{T}}{|}_{c=c_{k}}=\frac{\partial r_{k_{1}k_{2}}^{j}}{\partial F}\frac{\partial F}{\partial c^{T}}{|}_{c=c_{k}} \end{array}$$

Apart from the Jacobians with analytical or closed forms, the system actually needs a numerical solution to obtain the Jacobian of the dense depth map generated from the network with respect to the depth code $c_{k}$ according to the chain rule, i.e., $\frac{\partial F}{\partial c^{T}}{|}_{c=c_{k}}$. Although general deep learning libraries are able to calculate the gradient vectors among variables, it is really a time-consuming task to derive the full Jacobian matrices in the full dimension of [H,W,C] (*H*: image height, *W*: image width, *C*: depth code dimension) by general deep learning libraries because of keeping intermediate variables in the auto-gradient. For the purpose of computing the full Jacobian in real-time, the system performs a numerical method based on the parallel finite difference, leveraging the batch forward on GPUs to compute $\frac{\partial F}{\partial c^{T}}{|}_{c=c_{k}}$ [18]:

(17) $$\begin{array}{cc} J_{c_{k}}^{F} & =\frac{\partial F}{\partial c^{T}}{|}_{c=c_{k}}\\ \; & =\frac{\partial F(I_{k},S_{k},c_{k})}{\partial c^{T}}{|}_{c=c_{k}}\\ \; & \approx \frac{F\left(I_{k},S_{k},c_{k}+\delta c\right)-F\left(I_{k},S_{k},c_{k}\right)}{{\delta c}^{T}} \end{array}$$

where $\delta c\in R^{C\times 1}$ is the zero-approaching perturbation vector with the same dimension as the depth code $c_{k}$. The system can obtain the numerical approximate Jacobian fast by using Equation (17) rather than backward() and grad() operations in deep learning libraries. We implement the Jacobian computation module in a parallel way inspired by Ref. [18], based on the batch forward on GPUs but with a extremely lower dimensional depth code (depth code dimension C=8 in our method). Specifically, we can construct a 4D tensor γ with a dimension of [C,C,1,1] from repeating the original depth code *C* (in the shape of [C,1]) times in the batch size dimension. Then, for every depth code vector $c_{k}$ from γ in the batch size dimension, we only add the perturbation δ to one corresponding entry of $c_{k}$, respectively, and operate it to the tensor γ for every entry over the batch size dimension, respectively. Finally, we take γ as the batch input of our network and compute the full Jacobian $J_{c_{k}}^{F}$ in the dimension of [H,W,C], as in Equation (17). For the depth code Jacobian related to a particular pixel in Equation (13) or Equation (14), we can directly take the corresponding Jacobian vector of the pixel from the full Jacobian obtained above to the optimization solver.

**Figure 6:**
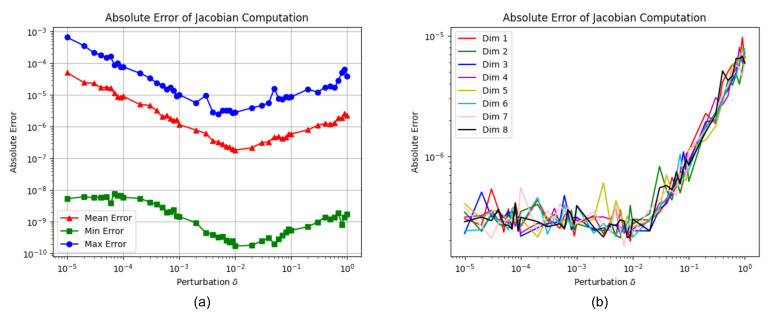
The absolute error of Jacobian computation between the proposed numerical method and auto-gradient functions in the deep learning library. (**a**) Comparison of the results with the proposed method and deep libraries by varying the perturbation δ from $1.0^{-2}$ to 1.0; (**b**) absolute Jacobian error for a certain dimension by varying the δ from $1.0^{-2}$ to 1.0, while fixing the δ for other dimensions.

To achieve an optimal perturbation δ for an accurate Jacobian estimation, a grid search experiment of δ from $1.0^{-5}$ to 1.0 with 600 images randomly collected from the NYU Depth V2 [20] and EuRoC [38] datasets was performed. In this experiment, we set δ equally for all dimensions of the depth code. The evaluation results are given in Figure 6a, where the X-axis is the search range of δ and the Y-axis is the absolute error between the proposed numerical Jacobian estimation and the deep learning framework. From Figure 6a, we can see that the optimal δ is around $1.0^{-2}$, which gives the most accurate numerical Jacobian estimation.

Furthermore, in order to validate the best δ for different dimensions in the depth code, we also test a range of δ from $1.0^{-5}$ to 1.0 for every dimension of the depth code (8D) while fixing the $\delta =1.0^{-2}$ for the other seven dimensions. As shown in Figure 6b, the average absolute error of Jacobian computation reaches the minimum around $\delta \approx 1.0^{-2}$ for all eight dimensions. Therefore, we fix $\delta =1.0^{-2}$ for all eight dimensions in the proposed method to compute the Jacobian numerically in experiments mentioned later. In addition, thanks to the shorter depth codes in the proposed network, the average time taken by Jacobian computation is about 4.5 ms on our machines (CPU: Intel Core i7-9700@3.0 GHz, GPU: NVIDIA GTX 1660Ti). It is obviously more fast than ~340 ms of the method using the deep library in DeepFactors [17] and ~10 ms of CodeVIO [18] with 32-dimensional depth codes under the close computation resources (NVIDIA GTX 1080 GPU in DeepFactors, NVIDIA GTX 1080Ti GPU in CodeVIO).

## 6. Experimental Results

The light-weight network described in Section 4 has been trained on the NYU Depth V2 dataset [20] with the official split of data and evaluated on NYU Depth V2 and EuRoC MAV [38] datasets for in-domain and cross-domain performance, respectively. The trained model was embedded into the visual-inertial SLAM system and we evaluated the trajectory accuracy of the visual-inertial dense SLAM system in the 6 indoor Vicon room sequences of EuRoC dataset. All evaluation experiments are conducted on the commercial desktop computer with an Intel Core i7-9700@3.0 GHz CPU and an NVIDIA GTX 1660 Ti GPU.

### 6.1. Evaluation of Depth Estimation on NYU Depth V2 Dataset

NYU Depth V2 dataset consists of RGB and depth images collected from 464 different indoor scenes with a Kinect RGB-D camera [20]. For training of the network, data augmentation was performed online with a sequence of random transformations [24]:

- Scale: input RGB/gray images are scaled by a random scale s∈[1.0,1.5] and corresponding input depth images are divided by *s*;
- Rotation: input RGB/gray and depth images are rotated with a slightly larger angle α∈[−10.0,10.0] in degrees to simulate the dynamic motion of sensor bodies;
- Color Jitter: input RGB/gray image brightness, contrast, and saturation are adjusted by a random factor $f_{color}\in \left[0.8,1.2\right]$;
- Horizontal Flips: input RGB/gray and depth images are synchronously flipped in the horizontal side by a probability of 50%.

Finally the input RGB/gray and depth images are cropped in center into the 224×224 resolution. In order to fit the front-end of SLAM systems, in the training phase of modalities with sparse depths, we detect FAST corners [39] on the RGB/gray images and randomly perturb the depth ground truths of these corners by introducing the noise under the normal distribution (std = 0.1 m) for the robustness to the inaccurate sparse point depths from the SLAM system in the online phase.

The evaluation of depth estimation for light-weight networks has been performed among multiple modalities and networks. With regard to light-weight networks for dense visual-inertial SLAM and embedded systems, the evaluation includes networks in FastDepth [23], CodeVIO [18], and this paper in 4 modalities (RGB, RGB + Sparse Depth, Gray, and Gray + Sparse Depth). Additionally, we also add the experimental results of our implementation of the network in CodeVIO with 32D depth codes (CodeVIO (32D)) and our implementation of the network in CodeVIO with 8D depth codes (CodeVIO (8D)) for ablation experiments about attention modules and the dimension of depth codes. Notice that both CodeVIO (32D) and CodeVIO (8D) do not contain any attention modules. In the evaluations of modalities with the sparse depth input, the sampled sparse depth number of FAST corners always keep 125 without noise for the comparison with networks in CodeVIO [18]. The error metrics used in the evaluation include: root mean squared error (RMSE), inverse root mean squared error (iRMSE), mean absolute relative error (Abs Rel), mean absolute error (MAE), mean absolute percentage error (MAPE), and the percentage of predicted image pixels within a threshold of relative error ($\delta_{1}$, $\delta_{2}$, $\delta_{3}$) [40]. We also report the inference time results of all methods (in millseconds: ms).

**Figure 7:**
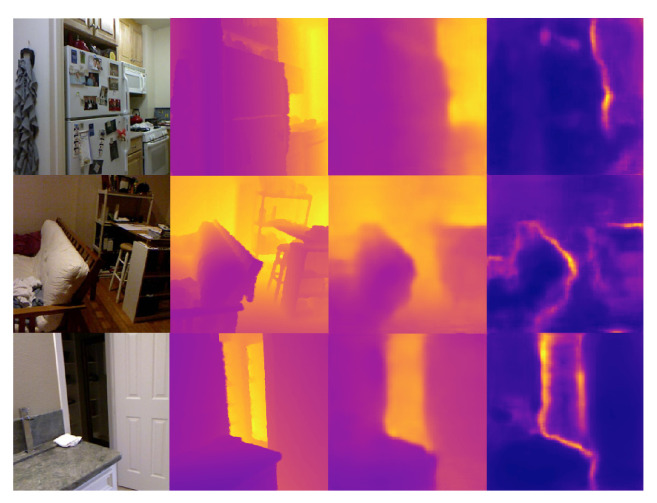
Some result visualizations of dense depth and depth uncertainty predictions on NYU Depth V2 dataset in RGB modality. From left to right column: input RGB images, dense depth ground truth, and predictions of dense depth and depth uncertainty maps.

**Figure 8:**
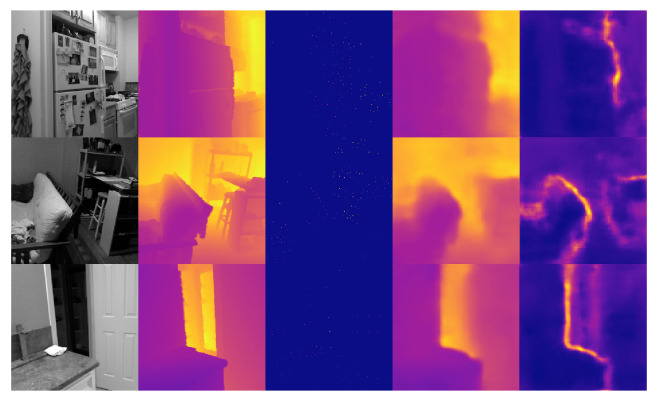
Visualization of dense depth and depth uncertainty prediction on NYU Depth V2 dataset in Gray + Sparse Depth modality. From left to right column: input gray images, dense depth ground truth, sampled sparse FAST corners, and predictions of dense depth and depth uncertainty maps.

Table 1 shows the evaluation results of depth estimation on NYU Depth V2 dataset. Over all input modalities (RGB, Gray, RGB + Sparse Depth, and Gray + Sparse), our method combined with attention modules (CBAM and SK-like attention) outperforms the other light-weight depth estimation networks embedded in dense SLAM by a large margin. Figure 7 and Figure 8 show some representative prediction results in RGB and Gray + Sparse Depth modalities on NYU Depth V2 dataset. Particularly, the results illustrate that depth estimation aided by sparse depth with the metric scale significantly promotes the prediction accuracy and generates more fine-grained results. This is suitable for visual-inertial SLAM systems, which maintain sparse maps with the metric scale. It is reasonable that the depth uncertainty tends to fit the region where gray scale and depth values change sharply. Notably, the dimension of implicit latent depth codes in our network is only 8 instead of 32 in the previous literature [18]. Although the dimension of depth codes in our method is 75% shorter than the other research [18], the depth prediction shows more accurate results with less memory usage, computation consuming, and inference time. This is actually important for robotic or embedded systems, which are concerned about the real-time performance on devices with limited computational resources.

### 6.2. Evaluation of Depth Completion on EuRoC Dataset

To evaluate the dense mapping performance of the proposed system, experiments of depth prediction evaluation have been also accomplished on all 6 indoor Vicon room sequences of EuRoC MAV dataset [38]. The EuRoC dataset consists of 6 indoor sequences in a laboratory equipped with the Vicon system and 5 sequences in a large machine hall. The dataset includes visual (from 2 cameras) and inertial (from 1 IMU) data collected by micro-aerial vehicles (MAVs). There is a LiDAR point cloud data of the indoor environment for every Vicon room sequence. We project the LiDAR point cloud onto every frame using the ground truth of frame poses and leave the minimum depth value at every local cell because of occlusions to render depth maps, which serve as the depth ground truth for each frame.

We evaluate the depth prediction of the proposed network in Gray + Sparse Depth modality only trained on NYU Depth V2 dataset for cross-domain performance and the results are shown in Table 2. We also evaluate the sparse depth accuracy of ORB points from ORB-SLAM3 system in monocular-inertial mode. Although the image feature from NYU Depth V2 and EuRoC datasets is quite different, the network only trained on NYU Dpeth V2 can estimate depth maps reasonably on EuRoC dataset and behaves with improved accuracy and generalization capability thanks to the attention modules and the input metric sparse depth. Therefore, the cross-domain generalization capability of the proposed method allows that we can only train the network on datasets collected by depth detection sensors (e.g., LiDAR, RGB-D cameras) with dense depth supervision and embed the trained model to a real-time SLAM system for tracking and dense mapping simultaneously.

**Figure 9:**
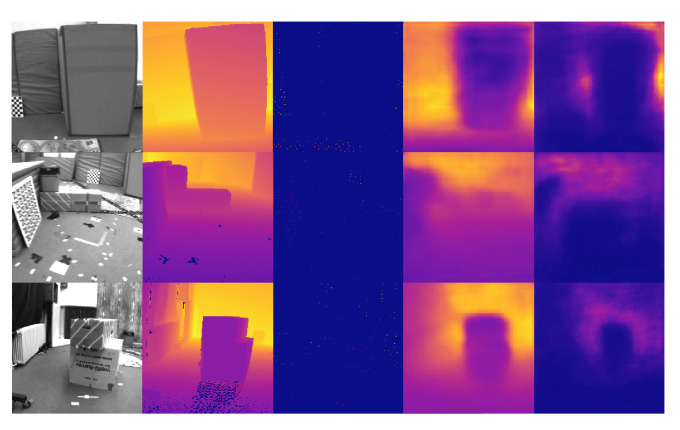
Cross-domain performance. Dense depth and depth uncertainty prediction results on EuRoC dataset in Gray + Sparse Depth modality of the network only trained on NYU Depth V2 dataset. From left to right column: input gray images, dense depth ground truth, sampled sparse FAST corners, and predictions of dense depth and depth uncertainty maps.

The evaluation results of dense depth on the EuRoC dataset with different methods are given in Table 2, including ORB-SLAM3 (Sparse ORB), CodeVIO from Ref. [18], our implemented CodeVIO with 32D depth codes (CodeVIO (32D)), our implemented CodeVIO with 8D depth codes (CodeVIO (8D)), and our proposed network. Figure 9 shows some results on the EuRoC dataset. It is notable that the networks of CodeVIO (32D) and CodeVIO (8D) do not contain any attention modules. Meanwhile, we include the results of CodeMapping from Ref. [19] to show our performance is comparable while our method is much faster. CodeMapping utilizes a more heavy-weight network and the number of input sparse points of CodeMapping in evaluation is 200–1000 per frame [19]. However, the number keeps 125 unchanged in our network and CodeVIO [18]. In addition, CodeMapping’s timing results were evaluated with a more powerful GPU, i.e., NVIDIA GTX 3080.

### 6.3. Evaluation of Trajectory Accuracy on EuRoC Dataset

**Figure 10:**
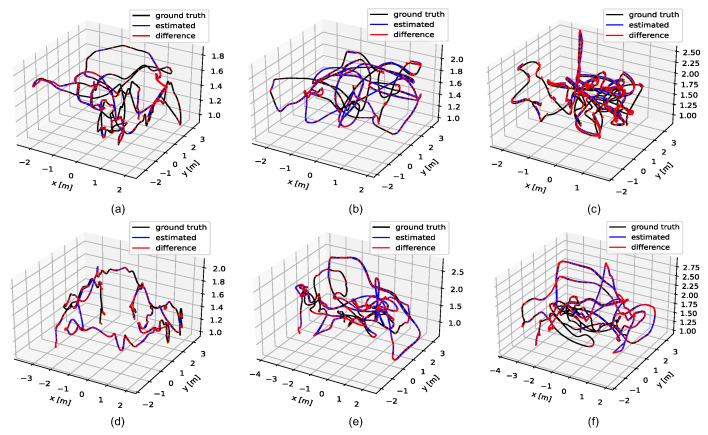
Trajectory results of the proposed system on 6 Vicon room sequences of EuRoC dataset. Trajectory illustration for sequences: (**a**) V101; (**b**) V102; (**c**) V103; (**d**) V201; (**e**) V202; and (**f**) V203.

All six Vicon room sequences of EuRoC dataset contain the 6D pose ground truth of sensor bodies recorded by a motion capture system. We also evaluate the trajectory accuracy in the 6 indoor sequences. Trajectory illustration compared with ground truth and RMS ATE [41] results are, respectively, shown in Figure 10 and Table 3. The trajectory results reported of ORB-SLAM3 were obtained by running the open-source code with its default configuration, and results of OpenVINS [42] and CodeVIO are from Ref. [18], since CodeVIO is not open-sourced, which is based on a particular version of OpenVINS.

**Figure 11:**
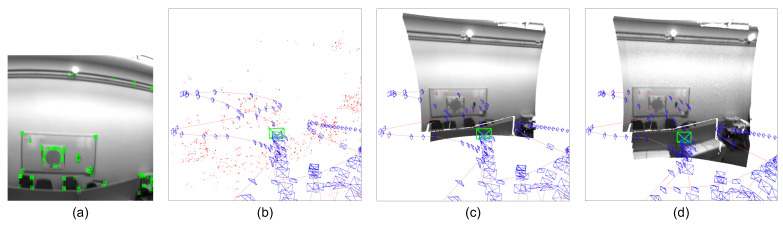
Textureless environments: a white wall. (**a**) The current 2D perspective with sparse feature points; (**b**) 3D sparse mapping from ORB-SLAM3; (**c**) 3D dense point cloud mapped from the current perspective; (**d**) 3D local dense mapping of the current sliding-window. There are few feature points on the white wall so that the sparse map generated from ORB-SLAM3 does not contain the information from the white wall. However, the depth completion network in the proposed system can predict dense depth maps so that the system is able to perform 3D local dense mapping, even in these textureless scenes.

**Figure 12:**
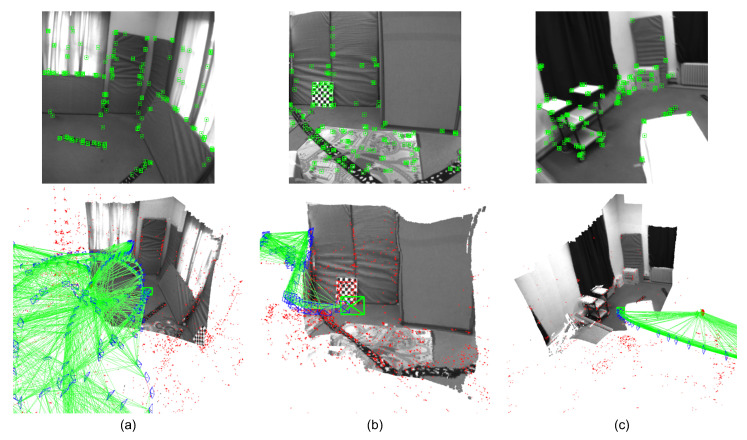
Demonstrations of tracking and local dense mapping on the EuRoC dataset. (**Top**): 2D feature tracking. (**Bottom**): 3D local dense mapping. (**a**,**b**): V1 sequences in the EuRoC dataset; (**c**): V2 sequence in the EuRoC dataset.

The results of trajectory evaluation indicate that tightly-coupled graph optimization on visual, inertial, and depth residuals has potential for the improvement of the localization accuracy, especially in textureless or featureless environments, such as a white wall (Figure 11). In Figure 11, there are few feature points on the white wall so that the sparse map generated from ORB-SLAM3 does not contain any information from the white wall. On the contrary, the depth completion network in the proposed system can predict dense depth maps so that the system is able to perform 3D local dense mapping even in these textureless scenes. It is useful for mobile robots to generate 3D local dense maps (e.g., local occupancy grid maps) for obstacle avoidance. Previous feature-based visual SLAM methods are prone to failure in featureless environments due to the lack of geometric constraints from rare feature matching. The depth estimation networks, however, can naturally provide geometric information and handle the rotation-only motion. Therefore, the depth consistency residual is designed for robustness in featureless scenes based on sampled points instead of feature points. In the sequences that contain more featureless periods (e.g., V102, V103), the proposed system estimates a more accurate trajectory. It benefits from the tightly-coupled optimization scheme. The results demonstrate that localization and dense mapping can promote each other. Particularly, the SLAM systems based on geometric methods are more likely to suffer from tracking failures in featureless environments. However, depth estimation networks can predict dense depth and provide geometry information and depth residuals of sampled points from featureless regions to guide the optimization on state and geometry estimation. This is quite crucial for visual SLAM frameworks. There are more demonstration examples of the proposed system for tracking and local dense mapping on EuRoC dataset in Figure 12.

## 7. Discussion

The proposed system can perform local dense mapping under the tightly-coupled graph optimization strategy, which takes advantage of the co-visibility graph managed by the sparse SLAM framework. Meanwhile, the co-visibility graph can provide not only the best local keyframes and states to be optimized, but also the best global keyframes [7]. Nevertheless, under the current system architecture, global dense mapping is a computationally intensive and time-consuming task. Consequently, this work proposes a dense SLAM system that targets the real-time local dense mapping. Specifically, we achieve the improvement of localization accuracy, considering the geometric constraints from the depth estimation under the tightly-coupled graph optimization, particularly in challenging scenes. In the future, an effective design of the keyframe database management and co-visibility graph for real-time global dense mapping combined with the proposed methods can be a research exploration.

## 8. Conclusions

In this paper, we have developed a real-time dense monocular visual-inertial SLAM system with the depth code-based representation of dense depth maps. In the proposed system, the state variables (e.g., poses and sparse maps) and the depth codes are optimized simultaneously in a tightly-coupled manner by considering visual, inertial, and depth residuals together. With this type of design, the accurate local dense geometry estimation can boost both the pose and map optimization. On the one hand, pose and sparse map estimation in the visual-inertial system can provide precise poses and sparse depth values with a metric scale to the light-weight network. On the other hand, the trained depth estimation network can predict the uncertainty-aware dense depth and guide the tightly-coupled optimization by introducing prior geometric constraints combined with the robust point sampling strategy, especially in textureless or featureless environments where the feature-based SLAM system is prone to tracking and mapping failure. Moreover, we evaluated the proposed network and system on two public datasets and presented that our methods achieved more accurate and robust results on both depth and trajectory estimation, even on the never-seen cross-domain dataset. The final experimental results on the public benchmark have verified the effectiveness of our system. Additionally, the proposed methods combined with global consistent dense mapping or other sensors can be taken as the future research directions.

## Figures and Tables

**Table 1:** Depth evaluation of typical light-weight depth estimation methods and our proposed network with different modalities (RGB (RGB), Gray (G), RGB + Sparse Depth (RGB-S), and Gray + Sparse Depth (G-S)) on the NYU Depth V2 dataset. The best is shown in bold.

Modalities	Methods	RMSE ↓	iRMSE ↓	Abs Rel ↓	MAE ↓	MAPE ↓	$\delta_{1}↑$	$\delta_{2}↑$	$\delta_{3}↑$	Time ↓
RGB	FastDepth ^1^	0.598	0.098	0.161	0.443	0.139	76.6%	93.9%	98.2%	2.498
	CodeVIO ^2^	0.545	0.090	- ^3^	-	0.130	83.0%	95.1%	98.5%	3.713
	CodeVIO (32D) ^4^	0.557	0.099	0.127	0.398	0.137	83.3%	92.0%	96.3%	3.932
	CodeVIO (8D) ^5^	0.582	0.110	0.144	0.401	0.150	81.8%	93.6%	96.2%	**3.480**
	Ours	**0.498**	**0.075**	**0.119**	**0.328**	**0.121**	**86.8%**	**96.6%**	**98.7%**	3.764
G	CodeVIO	0.535	0.089	-	-	0.133	83.3%	95.3%	98.6%	3.796
	CodeVIO (32D)	0.551	0.085	0.136	0.395	0.131	80.4%	92.3%	95.9%	3.782
	CodeVIO (8D)	0.587	0.116	0.147	0.397	0.141	83.2%	90.4%	94.5%	**3.237**
	Ours	**0.515**	**0.079**	**0.133**	**0.394**	**0.130**	**85.2%**	**96.1%**	**98.8%**	3.689
RGB-S	CodeVIO	0.316	0.052	-	-	0.066	94.4%	**98.6%**	**99.6%**	3.745
	CodeVIO (32D)	0.331	0.063	0.083	0.191	0.078	93.3%	98.2%	97.8%	3.940
	CodeVIO (8D)	0.337	0.075	0.094	0.202	0.075	93.1%	96.5%	98.1%	**3.547**
	Ours	**0.298**	**0.047**	**0.070**	**0.179**	**0.062**	**94.9%**	98.3%	99.3%	3.738
G-S	CodeVIO	0.315	0.055	-	-	0.071	93.9%	98.5%	**99.5%**	3.746
	CodeVIO (32D)	0.327	0.072	0.070	0.185	0.073	93.2%	98.2%	97.3%	3.792
	CodeVIO (8D)	0.343	0.075	0.081	0.191	0.090	91.8%	95.1%	98.4%	**3.403**
	Ours	**0.290**	**0.042**	**0.061**	**0.170**	**0.059**	**95.1%**	**98.9%**	**99.5%**	3.760

^1^ Results of FastDepth were from the model released by authors [23]. ^2^ Results were obtained from Ref. [18]. ^3^ denotes the item data and the source code of the corresponding method are not released by authors. ^4^ CodeVIO (32D) is our implementation of the network in CodeVIO [18] with the depth code in 32 dimensions. ^5^ CodeVIO (8D) is our implementation of the network in CodeVIO [18] with the depth code in 8 dimensions. The networks of CodeVIO (32D) and CodeVIO (8D) do not contain any attention modules.

**Table 2:** Depth evaluation of sparse depth from map points of SLAM (Sparse ORB) and light-weight dense depth estimation methods in the Gray + Sparse Depth modality on the 6 Vicon room sequences of EuRoC dataset. The best is shown in bold.

Sequence	Methods	RMSE ↓	iRMSE ↓	Abs Rel ↓	MAE ↓	MAPE ↓	$\delta_{1}↑$	$\delta_{2}↑$	$\delta_{3}↑$	Time ↓
V101	Sparse ORB ^1^	0.251	0.058	0.061	0.105	0.049	96.2%	98.7%	99.0%	-
	CodeMapping ^2^	0.381	-	-	0.192	-	-	-	-	11.00
	CodeVIO ^3^	0.468	0.091	- ^4^	-	0.107	87.0%	95.2%	97.9%	-
	CodeVIO (32D) ^5^	0.488	0.103	0.100	0.251	0.137	83.8%	93.1%	97.5%	3.794
	CodeVIO (8D) ^6^	0.503	0.134	0.132	0.283	0.154	81.4%	91.7%	95.3%	**3.412**
	Ours	**0.405**	**0.069**	**0.098**	**0.208**	**0.091**	**91.8%**	**95.7%**	**98.2%**	3.767
V102	Sparse ORB	0.380	0.074	0.089	0.167	0.088	92.4%	96.1%	98.0%	-
	CodeMapping	0.369	-	-	0.259	-	-	-	-	11.00
	CodeVIO	0.602	0.118	-	-	0.170	78.7%	91.9%	96.4%	-
	CodeVIO (32D)	0.621	0.123	0.113	0.285	0.173	76.9%	92.2%	97.0%	3.787
	CodeVIO (8D)	0.640	0.135	0.112	0.291	0.193	72.4%	90.0%	94.8%	**3.337**
	Ours	**0.511**	**0.103**	**0.109**	**0.266**	**0.117**	**90.1%**	**93.2%**	**97.2%**	3.758
V103	Sparse ORB	0.419	0.078	0.097	0.229	0.098	91.0%	94.7%	97.9%	-
	CodeMapping	0.407	-	-	0.283	-	-	-	-	11.00
	CodeVIO	0.687	0.103	-	-	0.198	73.9%	90.2%	96.5%	-
	CodeVIO (32D)	0.684	0.125	0.124	0.313	0.213	73.4%	90.1%	94.8%	3.790
	CodeVIO (8D)	0.693	0.149	0.130	0.337	0.223	75.9%	89.7%	94.0%	**3.414**
	Ours	**0.588**	**0.097**	**0.123**	**0.304**	**0.158**	**88.2%**	**92.1%**	**96.8%**	3.753
V201	Sparse ORB	0.388	0.071	0.110	0.193	0.101	92.3%	95.8%	97.3%	-
	CodeMapping	0.428	-	-	0.290	-	-	-	-	11.00
	CodeVIO	0.656	0.117	-	-	0.163	77.3%	90.8%	96.0%	-
	CodeVIO (32D)	0.667	0.126	0.129	0.335	0.173	76.0%	90.2%	95.7%	3.766
	CodeVIO (8D)	0.683	0.154	0.125	0.349	0.191	74.5%	90.1%	93.2%	**3.409**
	Ours	**0.577**	**0.096**	**0.118**	**0.301**	**0.150**	**88.3%**	**93.4%**	**96.5%**	3.785
V202	Sparse ORB	0.513	0.099	0.120	0.257	0.112	90.3%	92.9%	96.3%	-
	CodeMapping	0.655	-	-	0.415	-	-	-	-	11.00
	CodeVIO	0.777	0.125	-	-	0.206	72.0%	88.3%	94.9%	-
	CodeVIO (32D)	0.758	0.113	0.173	0.345	0.193	79.7%	88.2%	95.0%	3.732
	CodeVIO (8D)	0.783	0.136	0.181	0.347	0.207	73.8%	85.2%	90.5%	**3.389**
	Ours	**0.598**	**0.105**	**0.159**	**0.316**	**0.159**	**84.1%**	**91.3%**	**96.5%**	3.729
V203	Sparse ORB	0.473	0.070	0.109	0.248	0.104	90.9%	93.3%	97.2%	-
	CodeMapping	0.952	-	-	0.686	-	-	-	-	11.00
	CodeVIO	0.652	0.097	-	-	0.177	75.6%	92.5%	97.3%	-
	CodeVIO (32D)	0.637	**0.092**	0.140	0.351	0.173	77.0%	93.4%	97.1%	3.757
	CodeVIO (8D)	0.653	0.108	0.161	0.372	0.188	74.8%	91.5%	96.2%	**3.458**
	Ours	**0.585**	**0.092**	**0.122**	**0.306**	**0.156**	**86.0%**	**93.1%**	**97.6%**	3.776

^1^ Sparse ORB results were computed for sparse ORB points from ORB-SLAM3 system in monocular-inertial mode. ^2^ Results were obtained from Ref. [19]. Notably, the number of input sparse points of CodeMapping in evaluation is 200–1000 per frame. Besides, its time results were evaluated with a more powerful GPU: NVIDIA GTX 3080. ^3^ Results were obtained from Ref. [18]. ^4^ Denotes the item data and the source code of the corresponding method are not released by authors. ^5^ CodeVIO (32D) is our implementation of the network in CodeVIO [18] with the depth code in 32 dimensions. ^6^ CodeVIO (8D) is our implementation of the network in CodeVIO [18] with the depth code in 8 dimensions. The networks of CodeVIO (32D) and CodeVIO (8D) do not contain any attention modules.

**Table 3:** Trajectory evaluation of monocular visual-inertial SLAM systems with light-weight networks for local dense mapping on the Vicon room sequences of EuRoC dataset (RMSE of ATE in meters: m). Framerate results are in FPS. The best is shown in bold.

Methods	V101	V102	V103	V201	V202	V203	Mean
ORB-SLAM3 ^1^	**0.035**	0.013	0.030	0.043	0.016	0.019	0.026
OpenVINS ^2^	0.056	0.072	0.069	0.098	0.061	0.286	0.107
CodeVIO ^3^	0.054	0.071	0.068	0.097	0.061	0.275	0.104
Ours (Framerate)	0.036 (16.4)	**0.011** (16.2)	**0.022** (15.7)	**0.041** (16.3)	**0.012** (15.5)	**0.017** (15.1)	**0.023** (15.9)

^1^ Results were derived by running the released source code with the default configuration. ^1,2^ These methods are visual-inertial SLAM system with sparse mapping. ^2,3^ Results were obtained from Ref. [18] because CodeVIO is based on a particular version of OpenVINS.

## Data Availability

The data used in this paper can be found in [20,38].
